# Analysis of caregiver perspectives on patients with mucopolysaccharidosis II treated with pabinafusp alfa: results of qualitative interviews in Japan

**DOI:** 10.1186/s13023-024-03112-1

**Published:** 2024-03-07

**Authors:** Kimitoshi Nakamura, Norio Sakai, Mohammad Arif Hossain, Julie B Eisengart, Tatsuyoshi Yamamoto, Kazunori Tanizawa, Sairei So, Mathias Schmidt, Yuji Sato

**Affiliations:** 1 Department of Pediatrics, Faculty of Life Science, Kumamoto University, 860-0862 Kumamoto, Japan; 2 Child Healthcare and Genetic Science Laboratory, Division of Health Sciences, Osaka University Graduate School of Medicine, 565-0871 Osaka, Japan; 3 JCR Pharmaceuticals, 11-18 Kusunoki-cho, 659-0015 Ashiya city, Hyogo Japan; 4 Department of Pediatrics, University of Minnesota, 55455 Minneapolis, MN USA

**Keywords:** Mucopolysaccharidosis type II, Hunter syndrome, Caregiver-reported outcomes, Pabinafusp alfa, Treatment experience, Quality of life, Qualitative caregiver interviews

## Abstract

**Background:**

Mucopolysaccharidosis type II (MPS II), or Hunter syndrome, is a rare X-linked metabolic disorder predominantly affecting males. Pabinafusp alfa, an iduronate-2-sulfatase enzyme designed to cross the blood-brain barrier, was approved in Japan in 2021 as the first enzyme replacement therapy targeting both the neuropathic and somatic signs and symptoms of MPS II. This study reports caregivers’ experiences of MPS II patients receiving pabinafusp alfa through qualitative interviews.

**Methods:**

Semi-structured, qualitative interviews were conducted with caregivers at seven clinical sites in Japan using a semi-structured moderation guide (Voice of the Caregiver guide). Thematic analysis was applied to the interview transcripts to identify symptoms and health-related quality of life impacts at baseline, changes during treatment, and overall treatment experience.

**Results:**

Seven caregivers from 16 trial sites participated, representing seven children aged 8–18 years who had received pabinafusp alfa for 3.3–3.5 years at the time of the interviews. Data suggest a general trend toward improvement in multiple aspects, although not all caregivers observed discernible changes. Reported cognitive improvements included language skills, concentration, self-control, eye contact, mental clarity, concept understanding, following instructions, and expressing personal needs. Further changes were reported that included musculoskeletal improvements and such somatic changes as motor function, mobility, organ involvement, joint mobility, sleep patterns, and fatigue. Four caregivers reported improvements in family quality of life, five expressed treatment satisfaction, and all seven indicated a strong willingness to continue treatment of their children with pabinafusp alfa.

**Conclusion:**

Caregivers’ perspectives in this study demonstrate treatment satisfaction and improvement in various aspects of quality of life following therapy with pabinafusp alfa. These findings enhance understanding of pabinafusp alfa’s potential benefits in treating MPS II and contribute to defining MPS II-specific outcome measures for future clinical trials.

**Supplementary Information:**

The online version contains supplementary material available at 10.1186/s13023-024-03112-1.

## Background

Mucopolysaccharidosis type II (MPS II), or Hunter syndrome, is a rare X-chromosome inherited lysosomal storage disease with a highly heterogenous clinical presentation involving progressive, irreversible neurologic and somatic disease, and often a significantly shortened lifespan. Typically, clinical signs emerge in children aged two years or older [1] and may include cognitive impairment, bone and joint deformities, hearing loss, recurring infections, and vital organ dysfunction [1–4]. MPS II is caused by deficient activity of the lysosomal enzyme iduronate-2-sulfatase (IDS), which leads to the accumulation of heparan sulfate and dermatan sulfate; this triggers pathological processes that result in clinical signs. Today, MPS II is recognized as a spectrum disorder, ranging from severely neuronopathic forms to presentations with only mild neurologic manifestations [4–6]. 

Several global development efforts are being made to ameliorate the disease burden and improve the quality of life for individuals with MPS II and their families. Amidst these efforts is an important increased emphasis on the patient experience, as successful treatment should positively impact how patients feel, function and survive [7].

In Japan, current treatment options for MPS II include hematopoietic stem cell transplantation, intravenous somatic enzyme-replacement therapy (ERT) with IDS, a combination of intracerebroventricular (ICV) with idursulfase beta ERT and somatic ERT using IDS, and a brain-penetrant form of IDS to treat central and somatic signs and symptoms of the disease (pabinafusp alfa). However, these methods, particularly conventional IV ERT, are not fully effective in treating central nervous system (CNS) symptoms because the blood-brain barrier (BBB) prevents the IDS enzyme from reaching the CNS at therapeutic levels [8]. Consequently, progressive neurocognitive deterioration remains a significant concern for patients at the neuronopathic end of the spectrum, and their caregivers. Although ICV-ERT using IDS has been shown to bring about improvements, or at least stability, in the neurocognitive symptoms of neuronopathic MPS II, IV-ERT is still necessary to manage the somatic/peripheral symptoms effectively [9]. Moreover, ICV infusion requires injection of the enzyme through the scalp into a catheter device previously implanted under the scalp. Such administration entails a risk of causing meningitis through non-sterile injections, so extra precautions need to be taken when administering idursulfase beta via the device.

Pabinafusp alfa (IZCARGO®, or JR-141) was approved in Japan in March 2021 for the treatment of MPS II. Developed by JCR Pharmaceuticals, this fusion protein, which consists of anti-transferrin receptor antibody and IDS, is the first in MPS II to penetrate the BBB via intravenous administration, offering a new treatment to address both the somatic/peripheral and CNS symptoms of MPS II [10]. Along with the marked reduction of pathological accumulation of substrates in the cerebrospinal fluid as evidence of the efficacy of the drug against CNS symptoms, initial clinical trials showed notable behavioral improvements, such as resumed smiling, reduced behavioral outbursts, and heightened responsiveness [10, 11]; similar results were reported in subsequent trials conducted in Japan and Brazil [10–14]. Of the 62 patients involved, 89% experienced either stabilization or improvement in their neurocognitive impairment within 52 weeks of ERT [10–14]. The behavioral changes observed warrant further investigation [15, 16], as little attention has been given to these subtle but important changes in previous trials. Recent studies hence emphasize the need for patient-reported data and psychometric assessments of the MPS population, as well as a better understanding of treatment experience, unmet needs, and care preferences [6, 17, 18].

The aim of this study was to comprehensively examine caregiver-reported experiences in an extension clinical trial of pabinafusp alfa in Japan, and to assess the impact of MPS II on patients and families and the benefits of treatment through qualitative exit interviews based on a semi-structured moderation guide named Voice of the Caregiver (VoC).

## Methods

### Study design and development

Our qualitative study aimed to elucidate the experiences and perceptions of caregivers of MPS II patients undergoing treatment with pabinafusp alfa in Japan. We employed a semi-structured interview format, utilizing a moderation guide (VoC guide - see supplementary material) developed in English and subsequently translated into Japanese. This guide, developed by experts in MPS II neuropsychological assessment, consisted primarily of open-ended questions designed to capture a comprehensive and nuanced account of the caregivers’ experiences.

### Sites and participant enrollment

Invitations were extended to all clinical sites participating in the pabinafusp alfa extension trial to engage in exit interviews, and through this process, offer their caregivers the opportunity to contribute their observations in the VoC interviews. The consent process for these interviews was efficiently managed by the clinical staff at each site, with training and initiation protocols delivered remotely by dedicated liaison staff. To ensure confidentiality and data security, all patient and caregiver contact details were stored in an encrypted format and were subsequently deleted following the completion of the study to maintain privacy.

### Interview procedures

Interviews were conducted by a pair of Japanese interviewers, both females, who were contracted by a third party external to JCR. These interviews were carried out remotely, either via telephone or online platforms, each spanning approximately 90 min. The time frame for these interviews was between December 2021, and March 2022. All procedures received prior approval from the respective local ethics committees. To reduce thematic bias, the first part of the interview included only open-ended questions. Caregivers were asked to mention signs, symptoms, observations, or changes therein, etc., without the interviewer mentioning specific symptoms or themes. Only if the interviewee did not mention certain symptoms or impacts was the interviewer then asked in the second part to specifically inquire about signs and symptoms related to (a) somatic/motor function, (b) activities of daily living, (c) behavior/mood, (d) executive function, or (e) social/language skills. In addition to the open-ended questions, the VoC interview guide included Likert-scale items to further quantify certain aspects of the caregivers’ experiences. All audio recordings from the interviews were transcribed verbatim, de-identified, and subjected to rigorous quality checks to ensure accuracy and confidentiality before analysis.

### Analysis

A synthesis of thematic analysis [19] and elements of grounded theory [20] was employed to enable a holistic understanding of the qualitative data. This methodological approach adhered to the reflexive thematic analysis framework by Braun & Clarke [21] and followed the consolidated criteria for reporting qualitative research (COREQ) reporting guidelines [22], ensuring the transparency and rigor of the study. The initial codebook was developed from the interview guide, which was informed by our research questions and expanded to include emergent themes from the data. Throughout the coding process, we performed quality checks and inter-coder reliability assessments using MAXQDA 2020 (VERBI Software, Berlin, Germany), resolving any discrepancies through consensus to ensure a robust thematic analysis.

## Results

We use the terms “caregivers” to refer to the caregivers participating in the interviews and “patients” to refer to the children with MPS II under the care of the caregivers (all of the caregivers were in fact parents of the patients); the patients did not participate in the interviews.

### Patient demographics

Of the 16 study sites, seven agreed to participate, each enrolling a single caregiver. The remaining sites declined due to either their own disinterest, the disinterest of the caregivers, or concerns about imposing additional burdens on the caregivers.

The sample consisted of seven caregivers representing seven male children enrolled in pabinafusp alfa trials between August and November of 2018; the patients had started treatment between October and December 2018 when aged between 5 and 15 years (mean 9 ± 3.51). At the time of the interviews, the patients were aged 8 to 18 years (mean 12.1 ± 3.38) and had received treatment for periods of between 3.25 and 3.5 years. Five patients had the neuronopathic phenotype of MPS II, and two were classified as being toward the non-neuronopathic end of the spectrum, with one exhibiting some neurocognitive impairments [23]. Patient demographics and medical histories are presented in Table 1.

**Table 1:** Patient demographics and medical history

Distribution (*N* = 7 total caregiver participants)	
Patient age at the start of trial	
Mean (SD)	9 (± 3.51)
Range	5–15 Years
**Patient age at VoC interviews**	
Mean (SD)	12.1 (± 3.38)
Range	8–18 Years
**Patient Sex**	
Male	7 (100.0%)
**Caregiver relationship to patient**	
Parent	7 (100.0%)
**Patient medical history**	
Phenotype: neuronopathic vs. non-neuronopathic*	Neuronopathic (*n* = 5, 71.4%), Non-neuronopathic (*n* = 2, 28.6%)
Date of enrollment in phase 3 study	22 August 2018–22 November 2018
Pabinafusp alfa treatment start date	2 October 2018–27 December 2018

## Background and diagnostic journey

Discussing their children’s MPS II diagnostic journey, the caregivers shared their experiences of symptom presentation and diagnosis. Regarding the initial presentation of symptoms, the caregivers reported physical changes (*n* = 5) including body stiffness, abnormal head shape, difficulties in walking, ear infections, frequent fevers, developmental issues (*n* = 4), and organ involvement (*n* = 4) prior to diagnosis. Specific symptoms and other initial presentation-related information can be found in Supplementary Data Table S1.

Four caregivers indicated that their children were diagnosed at between 4 and 4.5 years of age, while the other three indicated earlier ages of between 7 months and 1.5 years. The diagnostic journey began primarily due to the caregivers noticing the aforementioned physical changes, developmental issues, and organ involvement, which prompted them to seek medical advice. Three caregivers consulted pediatric specialists and endocrinologists, and four mentioned additional examinations and confirmatory tests.

## MPS II experience prior to treatment with pabinafusp alfa

The caregivers recounted various symptoms at the beginning of the clinical trials, including developmental involvement, motor issues, joint/tissue involvement, general illness/infections, hernias, gastrointestinal and respiratory issues, seizures, and other organ involvement. Table 2 displays the main symptom categories and the frequency of reports.

**Table 2:** Symptoms reported by caregivers prior to the pabinafusp alfa trial

Symptom domains	Specific sub-domain concepts (if applicable)
Developmental/neurological involvement (*n* = 6)	•General observable cognitive development issues (*n* = 3) •Diagnosed brain function/cognitive disorders (*n* = 2) •Specific behavioral and developmental concerns (*n* = 1, each)
Motor issues (*n* = 3)	•Walking (*n* = 2) •Falling, grip problems, and movement difficulties (*n* = 1, each)
Joint/tissue involvement (*n* = 3)	•Joint deformation/stiffness (*n* = 3) •Connective tissue disease (*n* = 1)
General illness/infections (*n* = 3)	•Ear infections (*n* = 2) •Generally prone to illness (*n* = 1)
Hernias (*n* = 3)	•Hernias (*n* = 3)
Others (less frequently reported)	•Difficulty swallowing, heart valve regurgitation, difficulty breathing, seizures (*n* = 1 each)

The caregivers further elaborated on the effects of MPS II, particularly noting those connected with cognitive development and physical abilities. There were also some common reports of impacts on schooling, bathroom use, and social interactions. All seven caregivers recollected hospitalizations due to hernia surgeries, laboratory and radiographic examinations, heart-related complications, and ear infections prior to the clinical trial. Among the caregivers of children with the neuronopathic phenotype, all five highlighted significant developmental challenges.

Before enrollment in the clinical trial, the caregivers experienced negative emotions, such as pain, worry, self-blame, and doubts over the prospects of improvement. After the trial began, one caregiver expressed optimism, and another acknowledged the positive impact of knowing that treatment could prevent progression. The caregivers also referred to the effects of MPS II on their families, which included caregiver stress, impacts on daily activities and work, and social and family impacts. Additional descriptions of the effects of MPS II on the patients and their families are detailed in Tables 3 and 4.

**Table 3:** Impacts reported by caregivers prior to the pabinafusp alfa trial

Impact domains	Pre-trial, specific sub-domain concepts (if applicable)
Hospitalizations (*n* = 7)	•Hospitalizations (*n* = 7)
Cognition/development (*n* = 5)	•Communication issues (*n* = 3) •Lack of independence (*n* = 2) •Necessity for feeding support, a decrease in task proficiency, issues with comprehension, and lapses in word recall (*n* = 1, each)
Physical limitations (*n* = 5)	•Stopping running or playing due to heart condition (*n* = 2) •Difficulty walking (*n* = 2) •Bedsores, needing a wheelchair, difficulty sitting on the floor or kneeling, inability to use chopsticks, precautions needed against falls (*n* = 1, each)
Bathroom issues (*n* = 3)	•Inability to use bathroom alone/needing help (*n* = 2) •Diapers needed (*n* = 2) •Inability to express the need to use the bathroom, large urine volumes, issues with toilet training (*n* = 1 each)
Schooling (*n* = 3) & social (*n* = 3)	•Hyperactivity leading to an inability to remain alone, struggles with social interaction, frequent school absenteeism, and the necessity for caregivers to accompany children during school activities, as well as reported difficulties in the educational setting (*n* = 1, each)
Seizures (*n* = 1)	•Seizures (*n* = 1)

**Table 4:** Family impacts reported by caregivers, prior to the pabinafusp alfa trial

Family impact concepts	Caregiver descriptions prior to trial
Caregiver stress (*n* = 6)	Life is centered on the child’s treatment/care, food preparation, difficulty controlling seizures, and helping with bathing, mobility, dressing, or eating. Also includes long commutes to the hospital for treatment, with caregiving becoming progressively more difficult.
Activities of daily living/work impacts (*n* = 5)	Limitations on daily activities, difficulty obtaining childcare, and work-related impacts, including difficulties with work, difficulty finding jobs, inability to work, obtaining second jobs, or becoming self-employed.
Social & family impacts (*n* = 4)	Changes in need for communication, impact on siblings or other children, strains on the family due to caregiving, as well as an acceptance of the situation.

Regarding disease progression before the clinical trial, the caregivers observed worsening of joint deformity/stiffness, developmental regression, slow physical growth, accelerated diverse symptoms (Table 3), hernias, and CNS effects. The only significant behavioral issue reported was hyperactivity (*n* = 3).

Most of the caregivers decided to enroll their child in the pabinafusp alfa trial after consulting with health care providers who recommended switching from idursulfase and emphasized pabinafusp alfa’s potential effects on the neurological disease burden. They also took their experiences with previous idursulfase treatments into account, hoping for positive outcomes for their children or others through their participation in the trial. Overall, the impact of MPS II on their children (developmental delay, cognitive disease burden, physical and mobility limitations, limitations in daily living and school curriculum, frequency of infections and hospital stays, social interactions, feeding) and the associated caregiver stress (helping with daily living, food preparation, impact on siblings, impact on professional career, arranging hospital stays) were high and consistent with the literature describing the burden of the disease [1–4].

## Treatment-related experiences with pabinafusp alfa

The caregivers shared the observations and experiences they, their children, and other family members had after their children began participating in the pabinafusp alfa trial. All seven caregivers mentioned treatment-specific burdens, and four reported experiencing emotional impacts due to the treatment. One caregiver mentioned side effects, while another reported that treatment with pabinafusp alfa went more smoothly than previous treatments, which had caused allergic reactions. The caregivers also discussed their experiences with administering infusions, the effects of treatment on their families, and their own emotional states. One caregiver noted an increased understanding of clinical trials due to their participation.

Feedback from the caregivers emphasized the overall burden of participating in a clinical trial, highlighting the number of school days missed, general anxiety, and anxiety associated with such invasive measures as collecting cerebrospinal fluid, absence from work, financial burdens, the need to stay at hospitals overnight, and the impact on care for other siblings.

Detailed descriptions of the caregivers’ observations related to treatment with pabinafusp alfa are shown in Table 5. Describing their children’s condition after the onset of treatment, the caregivers frequently reported improvements in developmental/neurological symptoms, motor function/mobility, musculoskeletal/somatic/organ involvement, joint/hand function, and illnesses/frequency of infections. Improvements were noted in relation to school and social life, emotional state, and cognition or development-related changes, with none of the caregivers citing deterioration in these factors. Both positive and negative changes were reported regarding physical function and activities of daily living. Detailed descriptions of the changes cited after treatment with pabinafusp alfa started are provided in Tables 6, 7 and 8. Supplementary Figs. S1–S3 provide detailed comparisons.

**Table 5:** Treatment-related experiences of patients and their families

Treatment experience	Descriptions
Treatment-specific experiences of patients (*n* = 7)	Having to miss school, long waits in the hospital, not understanding what is going on, treatments becoming part of daily life
Emotional impacts of treatment on patients (*n* = 4)	Worrying about others, stress, enduring the collection of cerebrospinal fluid
Treatment experiences of family (*n* = 7)	*Positive experiences* (*n* = 3): comfortable waiting spaces, smoother or shorter treatments, coordinator providing support, progression to an easier experience *Negative Experiences* (*n* = 4): needing to leave work for treatments, financial burden, staying overnight in hospitals, long treatment sessions, difficulty seeing child suffer, concerns over radiation exposure
Family/sibling experiences (*n* = 3)	Family and siblings looking after the patient, siblings need to stay with other family members, caregivers attempting to spend more time with other children
Emotional impacts on family (*n* = 2)	Stress of caregiver burden (time and finances), uncertainty about the future

**Table 6:** Changes reported by caregivers after the start of the pabinafusp alfa trial

Symptom domains	Specific sub-domain concepts (if applicable)
Developmental/neurological	**Improved** (*n* = 5): speaking/language skills (*n* = 4, two probed), concentration (*n* = 3, one probed), facial expressions (*n* = 2), self-control (*n* = 2, probed), eye contact, proactivity, mental clarity, quicker understanding of concepts (*n* = 1 each) **Worsened** (*n* = 1): gradual decline in cognition and concentration (*n* = 1), self-control (*n* = 1, probed)
Motor functioning/mobility	Improved: ability to walk (*n* = 3, one probed) **Worsened** (*n* = 2): difficulty moving around (*n* = 2), balance issues, movement ability regressed but variable (*n* = 1 each, same participant)
Musculoskeletal/somatic/ organ involvement	**Improved** (*n* = 3): tongue size (*n* = 2), putting on additional muscle, previously enlarged organs decreasing in size, no progression in organ, heart condition, stools (*n* = 1 each) **Worsened** (*n* = 1): back issues, chest catching/locking up (*n* = 1 each, same participant)
Joint/hand function	**Improved** (*n* = 3): flexibility (*n* = 2, one probed), less stiffness/softer muscles, joints improved with rehab/massage (*n* = 1 each) **Worsened**: hand/wrist stiffness (*n* = 1)
Fatigue	**Improved**: fatigue (*n* = 1) **Worsened**: fatigue (*n* = 2)
Others	**Improved**: ear infections/illness frequency (*n* = 2), sleeping patterns (*n* = 2, 1 probed) **Worsened**: difficulty swallowing, seizures (*n* = 1 each, same participant)

**Table 7:** Changes in impact on patients reported by caregivers after the start of the pabinafusp alfa trial

Child impact domains	Specific sub-domain concepts (if applicable)
Physical function	**Improved** (*n* = 4): sleeping habits, decrease in falls, reduced snoring, ability to keep the mouth closed and breathe through the nose, exposure to germs, ability to move around, ability to ascend/descend stairs, kneel, athletic ability (*n* = 1 each) **Worsened** (*n* = 3): wheelchair required, difficulty walking, difficulty running, difficulty kneeling, difficulty sitting cross-legged, difficulty rising from sitting (*n* = 1 each)
Activities of daily living	**Improved** (*n* = 4): eating independently (*n* = 2, I probed), using bathroom alone (*n* = 2, one probed), able to use chopsticks (unable previously), interest in phones, interest in picture books, improvements in bike riding, switching from diapers to underpants (*n* = 1 each) **Worsened** (*n* = 2): ability to eat (*n* = 2 probed)
School/social life	**Improved** (*n* = 5): social interactions (*n* = 4, three probed), school life (*n* = 3, two probed), social independence, going to school daily (*n* = 1 each)
Emotional state	**Improved** (*n* = 3): calm/less anxiety (*n* = 3 probed), happier/normal, empathetic (*n* = 1 each)
Cognition/development	**Improved** (*n* = 2): self-expression, understanding speech, speaking ability, following directions (*n* = 1 each)

**Table 8:** Changes in impact on family reported by caregivers after the start of the pabinafusp alfa trial

Family impact domains	Specific sub-domain concepts (if applicable)
Caregiver burden	**Improved** (*n* = 2): child more independent and able to complete daily chores, easier to encourage the child to do things (*n* = 1 each) **Worsened** (*n* = 4): difficulty ensuring child swallows properly, help needed with dressing, cannot help when the child is experiencing side effects, difficulty grooming, child requires full support (*n* = 1 each)
Social/family impacts	**Improved** (*n* = 2): concerns about the child’s safety, family happier and healthier thanks to the child’s better health, family relief at subdued disease progression, the child being back in school, ability to understand the child’s speech (*n* = 1 each) **Worsened**: impact on siblings (*n* = 1)
Caregiver activities of daily living	**Improved** (*n* = 2): completing daily chores, ability to work from home (*n* = 1 each)

The caregivers reported only positive functional changes in their children after treatment started, citing improvements in communication skills/responses, and reduced hyperactivity. The most important perceived changes were cognitive improvements, establishment of routines, increased body strength, and fewer allergic reactions. Caregivers also noted improvements in bathroom independence, impulse control, sensation seeking, and sleep patterns. Supplementary Data Table S2 provides details of the changes observed in the families’ outlooks for the future.

When rating the overall change in their child’s condition since treatment started, one caregiver reported “very much improved,” three reported “a little improved,” and the remaining three reported, “no change” (*n* = 3). None reported worsening. In terms of the families’ quality of life, one caregiver reported “very much improved,” two reported “moderately improved,” one reported “a little improvement,” and the remaining three reported, “no change” (*n* = 3).

Positive changes observed by the caregivers as a result of treatment with pabinafusp alfa corresponded to their main concerns expressed prior to enrollment in the study: Among the most frequently observed positive changes were improved physical function, mobility, less stiffness, eating independence, improved language and expression skills, and more proactivity, along with several comments hinting at improved executive functioning and curiosity, improved sleeping patterns, more social interactions, and fewer infections/illness. Significant improvements in school functioning were mentioned by five respondents. A comparative analysis of symptoms and impacts pre- and post-treatment is shown in supplemental Figs. S1 and S2, respectively. Despite the burden of participating in the trial, all caregivers reported their desire to continue their children’s enrollment in the trial.

## Feedback on pabinafusp alfa treatment

When asked to rate their level of satisfaction with their child’s treatment with pabinafusp alfa on a scale ranging from “Very Satisfied” to “Very Dissatisfied,” five of the caregivers (representing both the neuropathic and non-neuronopathic phenotypes) expressed “Satisfied”. One with a child with the neuronopathic phenotype reported being “Neither satisfied nor dissatisfied” due to the child’s continued gradual decline in functionality, even though test results were becoming more stable. Another caregiver, whose child also had the neuronopathic phenotype, was “Dissatisfied” due to unmet expectations regarding study and site communications, long infusion time, and issues related to compensation.

Regarding their willingness to have their children continue treatment with pabinafusp alfa, all seven caregivers, including the two who expressed some dissatisfaction with the treatment experience, reported that they were “very willing to continue”. When asked about previous infusions their children had received, all seven caregivers reported idursulfase. All seven also said pabinafusp alfa would be their treatment of choice, attributing this preference to pabinafusp alfa’s effectiveness in slowing down disease progression, bringing about observable clinical improvements, and acting on the CNS symptoms. Selected quotes from the caregivers are presented in Supplementary Data Table S3.

## Caregivers’ suggestions for enhancing clinical trials of pabinafusp alfa

When asked to propose improvements for future studies involving MPS II patients to optimize their experience, the caregivers recommended reducing the time burden, refining the treatment modality, improving patient experiences at clinical sites, providing financial accommodations, and making efficacy adjustments. Their suggestions for future trials can be found in Supplementary Data Table S4.

When asked about any helpful information they wished they had known prior to joining the study, three of the caregivers reported no sense of lacking information and believed they had received adequate information at the beginning of the study, two said they trusted the doctors, and one felt that the health care providers had provided a thorough explanation of the treatment.

The caregivers (*n* = 7) also noted that they would recommend pabinafusp alfa treatment to other families caring for members with MPS II. While acknowledging that the initial treatment may be challenging, they emphasized the importance of remaining confident that things would improve. They also expressed hope that others would be aware of the burden placed on caregivers and their families by the disease and its treatment, but they pointed out the substantial benefits of pabinafusp alfa in improving cognitive and other disease outcomes.

## Discussion

The clinical manifestations of MPS II have been extensively described in the literature [1–5]. However, understanding the experiences of patients and caregivers remains a critical area for further research due to the tremendous impact and strain of MPS II on both [6]. This study addresses this gap through a qualitative approach, providing insights into the caregiver experience, which complements our clinical assessment tools. The scope of the survey was broadened to obtain information on the clinical trial experience, with caregiver responses indicating that such factors as the time burden, experiences at the clinical sites, and financial needs should be addressed to alleviate the burden of trial participation.

Caregivers in this study reported tangible improvements in their children’s signs and symptoms and quality of life after enrolling in the pabinafusp alfa clinical trial. Caregivers of children with the neuronopathic phenotype of MPS II reported improvements in cognitive, developmental, and physical aspects, suggesting the potential of pabinafusp alfa in treating the neuropathic aspects of MPS II. The caregivers’ accounts encompass the physical, behavioral, and psychological experiences that impact both their own and the patients’ lives. In highlighting the caregiver experience, such observations and are important considerations in evaluating the effectiveness of pabinafusp alfa in treating MPS II.

While the sample size of this study was limited, it was reflective of a diverse range of experiences within the cohort treated with pabinafusp alfa. The inclusion of caregivers from multiple clinical sites across Japan provides a broad perspective on the treatment’s impact. However, we acknowledge the potential for variability in treatment outcomes and caregiver experiences, suggesting the need for further studies to explore these aspects more comprehensively.

Early diagnosis and initiation of ERT are vital factors in the treatment of individuals with MPS II [24, 25]. In this study, three of the seven patients were diagnosed early and given conventional ERT with idursulfase. However, persistent neurocognitive decline highlighted the need for alternative treatment options addressing the central nervous system aspects of the disease. In a previous study, pabinafusp alfa, was shown to maintain normal growth and development in a child treated early, in contrast to a sibling treated with idursulfase [26].

The caregivers in this study reported a wide range of MPS II symptoms in their children, from the time of diagnosis to the present. These symptoms significantly impacted the daily activities and overall well-being of their families, as they necessitated frequent hospitalizations that imposed social and emotional strain. After enrolling in the pabinafusp alfa clinical trial, the caregivers noted marked improvements in their children’s symptoms and quality of life. Caregivers of patients with the neuronopathic phenotype, who initially reported cognitive/developmental challenges, observed cognitive, developmental, and physical improvements in their children.

Some caregivers reported no change or deterioration in their children’s condition, reflecting the complexity of MPS II as a progressive disease affecting multiple organs. Despite this, all caregivers said they intended to continue pabinafusp alfa treatment, with most expressing satisfaction with the results.

For this qualitative study, the cohort of amenable subjects was 22 from 16 sites in Japan. Of these, seven caregivers (32% of the participants) from seven sites (44% of the active sites) consented to participate in the VoC interviews. Limited interest or capacity to engage by some clinical sites was a challenge. The small sample of caregiver participants from the trial is a limitation to the significance of the study. A larger sample might have been achieved if the VoC interviews had been incorporated into the trial protocol from the outset, rather than being added to the ongoing extension study protocol. Currently, over 70 patients are being treated with IZCARGO® (pabinafusp alfa). As participation in the VoC interviews was voluntary, the results may be subject to selection bias.

The caregiver-reported data may also have been influenced by recall bias, as the caregivers were asked to recall their child’s medical history and symptoms prior to treatment, which may have been several years before the interviews. Some caregivers, particularly those of the children with an earlier onset of symptoms, may not have accurately recalled all relevant symptoms and impacts throughout the long clinical courses. Another potential source of bias could have been the level of enthusiasm on the part of the caregivers to participate in the pabinafusp alfa clinical trials and persist with the treatment. This could have led them to either overstate or understate the relevant observations. Further, the sample of respondents was probably weighted towards those in favor of the trial. Some of the mentioned symptomology such as organ involvement (in particular organomegaly or liver function as diagnosed by blood analysis) requires more sophisticated diagnostic and analytic methods. Comments made by caregivers during interviews about changes were likely influenced by their conversations with the treating physician.

We recognize the inherent limitations associated with the retrospective recall of treatment experiences by caregivers. To mitigate the impact of recall bias, the study design included semi-structured interviews that focused on specific aspects of the treatment experience and its outcomes. Nonetheless, the potential influence of retrospective recall on the reported experiences cannot be entirely excluded.

It is also important to consider the ages of the study patients at the start of the trial (age 9 ± 3.51) when interpreting the results. Those aged six years or older presenting with early progressive or neuropathic forms of MPS II may differ from younger patients in terms of treatment responses. In older patients, disease stabilization rather than amelioration is generally accepted as the therapeutic goal, as demonstrated in natural history studies [27, 28].

Comparisons between the VoC interview results and other observer/clinician-reported outcome instruments, such as the Vineland Adaptive Behavior Scales (VABS), might be helpful in determining the degree of challenge to the child’s independence, which might provide context for some of the caregiver or family stresses. Unlike VABS, the VoC tool is useful in that it collects detailed data on disease progression and treatment course, thus capturing changes not reflected in the VABS that may be adaptable for other MPS subtypes. Future studies employing VoC to examine both prospective and retrospective data could be further fortified by incorporating more comprehensive outcome measures, such as the Clinical Global Impression and Patient Global Impression, which would enhance the robustness of the results.

Participation in a clinical trial, especially one involving a novel therapy like pabinafusp alfa, may inherently influence caregivers’ perceptions and experiences. Increased healthcare interactions and the novel nature of the treatment might contribute to more positive outlooks. It is important to consider this context when interpreting the findings of this study, as the experiences of caregivers and patients outside the clinical trial setting may differ.

## Conclusion

This pilot study provides insights into the experiences of caregivers of patients with MPS II, including their observations on the manifestations of the disease, and the effectiveness of treatment with pabinafusp alfa in improving quality of life. By highlighting the family experience during clinical trials and the daily challenges faced by patients with MPS II and their caregivers, the findings may also contribute to future clinical trial designs. The interview results may additionally aid in subgroup analyses stratified by clinical severity and treatment initiation stages.

### Electronic supplementary material

Below is the link to the electronic supplementary material.

**Additional Files** Supplementary Data for VoC MPSII Treated with Pabinafusp Alfa: includes referenced tables and figures

## Data Availability

The datasets generated and/or analyzed during this study are not publicly available due to the confidential nature of the patient data and ethical considerations. The data involves sensitive information from caregivers of patients with MPS II and is subject to privacy restrictions. However, the data is available from the corresponding author, upon reasonable request and subject to compliance with the appropriate privacy and confidentiality measures. Data are located in controlled access data storage at JCR Pharmaceuticals Ltd.

## Abbreviations

BBB: blood-brain barrier
CNS: central nervous system
ERT: enzyme replacement therapy
MPS: II mucopolysaccharidosis type II
VoC: Voice of the Caregiver
VABS: Vineland Adaptive Behavior Scales
