# Supplementary material for: Analysis of caregiver perspectives on patients with mucopolysaccharidosis II treated with pabinafusp alfa: results of qualitative interviews in Japan
Source: Orphanet J Rare Dis. 2024 Mar 7;19:104. doi: 10.1186/s13023-024-03112-1 (PMC10921713; doi:10.1186/s13023-024-03112-1)
Supplement: Supplementary file 1 — Additional Files Supplementary Data for VoC MPSII Treated with Pabinafusp Alfa: includes referenced tables and figures [file 13023_2024_3112_MOESM1_ESM.docx]

**Analysis of Caregiver Perspectives on Patients with Mucopolysaccharidosis II Treated with Pabinafusp Alfa: Results of Qualitative Interviews in Japan**

***Supplementary data***

Table S1: Initial Presentation of Symptoms

| Symptom domains | **Specific concepts (if applicable)** |
| --- | --- |
| Physical changes (n=5) | Body stiffness (n=2), head shape (n=2), issues with walking (n=2), ear infections (n=2), fevers (n=2), falling down (n=1), body feeling heavier (n=1) |
| Developmental issues (n=4) | Delayed speech (n=3), physical development issues (n=3), hearing issues (n=1) |
| Organ involvement (n=4) | Heart (n=2), liver (n=2), spleen (n=1) |
| Other reported symptoms | Excessive growth (n=1), slow growth (n=1), bone & joint deformities (n=1), hernias (n=1), respiratory symptoms (n=1) |
| Selected caregiver quotes |  |
| “My child suffered from many middle ear infections and often had a runny nose. And, when I would hug him, his body felt heavy – heavier than you would expect from his body weight. His body also felt stiff, and his head seemed a little misshapen: his forehead seemed to protrude a little.”  “I noticed his speech was delayed. Also, I noticed he did not hear well, but a doctor told me it was common for small children not to pay attention.”  “My child often fell down and had many symptoms that were a little… different: delayed physical development, and delayed language development.”  “The doctor mentioned his liver and spleen were enlarged and suggested getting some tests done because it might be a serious issue.”  “His blood work revealed that his liver function results were eight times higher than the normal range, so they telephoned us immediately and recommended we schedule an appointment with the pediatrics department for more tests.”  “I noticed his fingers were bent and didn't extend easily.” | |

Figure S1: Comparison of symptoms prior to the pabinafusp alfa trial and changes since the trial began

Figure S2: Comparative analysis of patient impacts on patients pre- and post-initiation of the pabinafusp alfa trial

Figure S3: Comparison of impacts on families prior to the pabinafusp alfa trial and changes after the trial began

Table S2: Family outlook and planning for the future

| Changes | **Descriptions** |
| --- | --- |
| Family outlook (n=4) | Improved emotional impacts of MPS II (n=2)  Necessity for favorable positive changes to support child’s future (n=1)  Worsening emotional impacts of MPS II due to progression (n=1)  Resigned to the situation (n=1) |
| Planning for the future (n=2) | Actively planning for the future (n=6)  Remaining hopeful, despite prevailing worries, without the need for a substantial improvement in future expectations (n=4)  Improved outlook (n=2)  Made changes to accommodate child’s needs (n=1) |
| Selected caregiver quotes |  |
| “To date, the disease has been quite difficult, and that’s all we were thinking about. However, I now feel we are thinking more about the future. The junior high school had a job experience field trip to a supermarket, where the kids did various things like stocking shelves, and he really seemed to enjoy it. I actually went and had a quick look and saw he was able to correctly stock the shelves. I thought, wow he is able to do this now as well.”  “I hope we can continue the treatment and he can maintain his current condition. I hope he can be taken care of by the facility after we are gone.”  “So, I am hopeful that my son will live into adulthood… And, now we are able to be more serious about teaching him things that he will need to be able to do when he is an adult or, for example, over the next 10 years… You know, he will still have this disease, but I hope he will be able to make it in such and such groups, achieve such and such goals, continue to see improvements in such and such activities, etc.”  “Well, for this current treatment they tell me that he will be at the current hospital for a further 10 years, so I’m hoping that it will be possible to receive the treatment at a hospital closer to home. That would mean treatment would only require half a day, and the stress levels would go down further, and the burden on us would also go down… I’m hoping that one day we will be able to live with the disease rather than having to fight it every day… it will become a part of regular life…” | |

Table S3: Feedback on pabinafusp alfa treatment

| Topic | **Selected caregiver quotes** |
| --- | --- |
| Overall satisfaction | “He no longer suffers any allergic reactions, and he is able to do more things, so I feel satisfied with the treatment.”  “I am satisfied because our lives have become more stable, and I expect this will continue.”  “For me, the fact that the disease is not progressing makes me feel very happy. The fact that with one visit to the hospital per week the disease has stopped progressing and he is now in a stable condition has been a great emotional boost for me. I’m glad that medical science has come so far.” |
| Pabinafusp alfa vs. idursulfase | It was hard to commute to the hospital weekly [for treatment with idursufase], especially since I had to take two hyperactive sons with me. For this reason, the current treatment is incomparably better.  There are pros and cons. Elaprase didn’t reach the central nervous system, but the treatment time was shorter. The medicine he’s using now takes more time. I would stay with the current one because it targets the central nervous system.  “I think the improvements with the current medicine are much more pronounced overall.”  “They did say that this new medicine would have a greater effect on development, so when we heard that, we were hopeful… We read that with his condition he would gradually lose his ability to speak, but he is speaking more than ever now and his mood is bright… And we also read that this new medicine would help to increase his lifespan.” |
| Willingness to continue | “I really want to continue… I feel that [pabinafusp alfa] is helping to slow down the progression of the disease.”  “I want to continue because [pabinafusp alfa] reaches the central nervous system and has the greatest efficacy.”  “Well, as I mentioned before, because of the improvements, we thought it was a good idea to continue.”  “Well… (given the improvements, we can’t stop, so) the only thing to do is continue… I hope that in the future they are able to improve its [pabinafusp alfa’s] effectiveness… At this point, I have no new concerns, and if there is improvement in my son, that is enough.” |

Table S4: Suggested improvements for future trials with pabinafusp alfa

| Suggested changes | **Descriptions** |
| --- | --- |
| Reduced time burden | Shorter duration of infusions (n=3), less frequent treatments (n=2), shorter waiting time before starting treatments (n=2), reduced travel time to hospital or treatment options closer to home (n=1) |
| Improve treatment modality | In-home treatments (n=1), changing treatment to pill form (n=1) |
| Improve site experience | Provide written data to participants (n=2), opportunities to discuss future planning with other families (n=1), more nurses who are better skilled at infusions (n=1) |
| Financial accommodations | Full financial support for all patients (n=1), hospital fees covered during treatment (n=1), tax-free status for study compensation (n=1) |
| Efficacy | Better efficacy in treating joints (n=1) |

**“Voice of Caregiver” Sub-Study Interview Guide (VoC Guide)**

**Study Title:** An Extension Study of JR-141 in Patients with Mucopolysaccharidosis Type II

**Study Sponsor:** **JCR Pharmaceuticals Co., Ltd.**

**PROTOCOL NO. JR-141-302**

**Thank you for speaking with me today. I am interested in learning about your experience as a caregiver of someone with MPS II, or Hunter Syndrome, as well as your experiences in the JR-141 clinical trial. During our interview, I will ask you some questions about:**

- Your son’s diagnosis of MPS II;
- Your son’s condition before the clinical trial;
- Treatment experience, including changes experienced during the clinical trial;
- Your thoughts about the treatment your son received;
- Experiences with the clinical trial not specific to treatment.

**There are no right or wrong answers to these questions. I am here to learn about you and your child’s experiences. You do not have to talk about anything you do not wish to talk about; please feel free to stop the discussion at any time.**

**Please note that I am a qualitative researcher and not a medical expert. If you have any questions you have about the condition or treatment, please contact your doctor.**

[Interviewer: determine patient’s relationship with interview participant, and whether they would prefer to refer to them as “my child” or “my son”; refer throughout.]

[Interviewer: participant may refer to JR-141 as “IZCARGO”; use whatever term the participant prefers.]

**As this interview will take up to 90 minutes, we can take a quick break during it. Please let me know at any point if you would like to take a break.**

**I will audio-record the discussion with your permission. The recording will be used to develop a transcript. To protect your confidentiality, I will use your study participant number instead of your name.**

**Do you have any questions before we begin?** [Pause and assess readiness to continue.]

**I will now start recording.** [Make sure recorder is on.]

**This is the JR-141-302 study Voice of Caregiver interview, the date is [state date], and I am speaking with participant number [state trial subject ID].**

**Can you confirm that you agree to have this interview audio-recorded?** [If “NO,” stop the recording; end the interview.]

**WARM-UP SECTION - BACKGROUND/DIAGNOSTIC JOURNEY [7-10 Minutes]**

1. I would like to start by confirming how you refer to your son’s condition? [Use the caregiver’s term for MPS II / Hunter Syndrome throughout the discussion, listen/probe for terms to characterize the condition.]
   1. When was your son diagnosed with MPS II? Listen/probe for referral to specialists, genetic testing, etc.]
   2. When did you start noticing that something was wrong or different? [Listen/probe for signs and symptoms such as physical changes, respiratory symptoms, organ involvement, hernias, slow growth, bone/joint deformities.]

**UNDERSTANDING EXPERIENCE *BEFORE* CLINICAL TRIAL [10-15 Minutes]**

1. I would like to know more about your son’s MPS II experience before starting JCR’s clinical trial.
   1. Why did you decide to enroll your child in the clinical trial?
   2. Please describe your son’s condition when you joined the study. [Listen for symptoms and impacts, behavioral issues, progression of disease, time spent in hospital, etc. If helpful, remind the caregiver of the date they entered the study. For example: “When your son entered the study in [month/year from patient information], what were his symptoms like? How was his life affected by MPS II?]
   3. Can you describe the most concerning symptoms he was experiencing at this time? [If participant did not already mention symptoms in the previous prompt.]
   4. How did your son’s MPS II affect the way you felt? [Listen/probe for emotional impacts.]
   5. How did your son’s MPS II affect your family’s life at this time? [Listen/probe for other disease impacts, caregiver burden, changes in activities of daily living, impacts to social/family relationships, etc.]

**CHANGE OVER TIME DURING CLINICAL TRIAL [25-30 Minutes]**

1. As part of the study, your son receives a series of infusions of the JR-141 treatment.
   1. What is the experience of receiving the treatment like for your son? [Listen for and note any adverse events (AEs). Report as required following the interview.]
   2. What is the experience like for you and your family? [Listen/probe for potential changes in disease status between first/last infusions.]

**[Interviewer note: For Q4-7 below, refer to the MPS II worksheet to note spontaneously reported concepts, and to review the list of specific concepts asked in Q7.]**

1. I’d like to focus next on your son’s health now. [Listen/probe for changes in symptomology, demeanor, and behavior if not mentioned previously.]
2. Since starting the infusions in [month, year], what changes have you noticed in your child? [Note each change. Probe for any other changes before proceeding to the next question to explore each one in more detail.]
3. I’d like to get some more detail about each change your son experienced [Ask 6a & 6b for each change mentioned.]
4. How did [restate symptom/impact/behavior] change?
5. Compared to when the infusions began, would you say that [restate symptom/impact]…
   - 1. significantly improved?
     2. moderately improved?
     3. improved at first, but then got worse?
     4. did not change?
     5. worsened?
     6. Or would you describe the changes in another way?

[Potential follow-up probes as appropriate]

1. *For symptoms:*
2. When did you notice the change in [restate symptom]?
3. [Probe about frequency, duration, severity of symptom, other ways that the symptoms changed.]
4. *For impacts on daily life or activities:*
5. When did you notice the change in [restate impact/activity]?
6. Has there been a change in how well he can do [restate activity]? [Probe about frequency & length of activity, probe to get number of times per week/month, as appropriate.]
7. Have there been any changes in [restate activity] that you need assistance with? [Probe: description of change and reasons for the changes in activity]
8. Are there other ways that [restate activity/impact] has changed?
9. *For behavioral changes:*
10. When did you notice the change in [restate behavior]?
11. How has this change in your son’s behavior impacted your daily life? How about your family as a whole?
12. **[If participant DOES NOT spontaneously mention the following symptoms/impacts, ask the relevant questions below using specific probes; refer to the MPS II worksheet. Do not ask about concepts that have already been reported in Q6.]**
13. **[Somatic/Motor]** Has your son experienced any changes in…
    1. pain or discomfort?
    2. fatigue?
    3. hand function? [handwriting, using chopsticks/eating utensils, gesturing]
    4. strength? [if there is a prior history of weakness]
    5. walking or mobility?
    6. seizures? [only if patient has a history of seizures] Any changes in frequency or type?
14. **[Activities of Daily Living]**
    1. Since beginning the infusions, how have these changes affected your son’s daily life, such as the activities he can do?
    2. Has your son gained any new skills that he was not able to do before?
    3. Have there been any changes in your son’s eating habits? [Any changes in the need to assist your child?]
    4. Have there been any changes in the way your son uses the bathroom? [Any changes in the need to assist your child?]
    5. Have there been any changes in your son’s sleeping habits?
    6. [Follow-up probe for each] How so? Can you provide an example?
15. **[Behavior/Mood]** Have you noticed any changes in your son’s…
    1. general behavior?
    2. mood? stress or anxiety level?
    3. level of activity [potentially addressed in Q7b above]
    4. [Follow-up probe for each] How so? Can you provide an example?
16. **[Executive Function]** Have you noticed any changes in your son’s…
    1. ability to pay attention or focus?
    2. ability to understand directions? ability to follow directions?
    3. ability to control himself [or, exercise self-control]?
    4. [Follow-up probe for each] How so? Can you provide an example?
17. **[Social/Language]** Have you noticed any changes in your son’s…
    1. interest in social interaction? [speaking and interacting with friends/other children, with strangers, with family]
    2. self-expression?
    3. use of language to communicate? [to the extent possible]
    4. experience with school? [for school age children]
    5. [Follow-up probe for each] How so? Can you provide an example?
18. You told me that you have noticed the following improvements in your son since the beginning of the study: [review list of all mentioned improvements].
19. Of all the improvements you have noticed:
20. which change do you think is the most important to your son?
21. which is the most important to you?
22. which is the most important to your family? [May be the same answer as above]
23. What are the reasons it is the most important (meaningful) to you (your family, your son)? What effects does it have on your/their daily life?
24. Which improvement is the next most important?
25. What are the reasons it is important (meaningful) to you?
26. [Have participant rank up to 3 improvements in order.]
27. **Overall**, which of the following best describes the **changes in your son’s condition** since the start of treatment?
28. Very much improved
29. Moderately improved
30. A little improved
31. Neither improved nor worsened
32. A little worsened
33. Moderately worsened
34. Very much worsened
35. Has the **quality of life of your family** changed as a result of your son’s infusion treatments, compared to when the study started? (If yes, how?) [Listen for areas of improvement and other changes. Probe where necessary to characterize each change.] Overall, which of the following best describes the changes in *your family’s* quality of life?
36. Very much improved
37. Moderately improved
38. A little improved
39. Neither improved nor worsened
40. A little worsened
41. Moderately worsened
42. Very much worsened
43. How has your and your family’s outlook for the future changed as a result of your son’s treatments/changes? [Probes: planning for the future, life expectancy, future in social settings (work, school, etc), level of need for assistance/caregiving]

**CLINICAL TRIAL – FEEDBACK ON TREATMENT OVERALL [10-15 Minutes]**

1. Overall, how satisfied or dissatisfied are you with the use of JR-141 to treat your son’s MPS II?
2. …Would you say that you are:
3. very satisfied?
4. satisfied?
5. neither satisfied nor dissatisfied?
6. dissatisfied?
7. very dissatisfied?
8. What are the reasons you chose X?
9. Is there anything about the treatment you would like to see improved? What is the reason you would make that change?
10. Before starting the JCR trial, did your son previously receive other infusions or treatments for his MPS II? [If yes] Which treatment did he receive? [Interviewer: If **Elaprase**, ask Q15a-c; if a **different treatment**, only ask Q15d; if NO **(previous treatment/treatment naïve)**: skip Q15]
11. How does your son’s experience with the JR-141 treatment compare to his previous treatment?
12. What was the Elaprase treatment like for you and your family?
13. Comparing your experience with the two treatments, which treatment do you prefer? [If clarification is needed: JR-141 or Elaprase?]
14. What are the reasons you prefer [restate preferred treatment]?
15. [Other previous treatments, if not Elaprase] What was your son’s previous treatment?
16. What was your experience like with [restate past treatment]?
17. Do you prefer the current JR-141 treatment or [restate past treatment]? Why?
18. Looking ahead, we would like to know if you plan to continue treatment with the JR-141 infusions. Would you say that you are:
    - 1. very willing to continue?
      2. somewhat willing to continue?
      3. somewhat unwilling to continue?
      4. not at all willing to continue?
19. What are the reasons you chose X?
20. Do you have any (additional) concerns about your son continuing to receive the treatment?

**JCR TRIAL EXPERIENCE [5-10 Minutes, OPTIONAL — IF TIME ALLOWS]**

1. Are there any improvements you would suggest for future studies with MPS II patients to make the experience better?
2. Is there any information you wish you had known before the study, to make the experience better?
3. Are there other experiences you have had as a caregiver in this clinical trial that might be important for us to know?

**CLOSING [5 Minutes, OPTIONAL — IF TIME ALLOWS]**

1. Is there anything more about your experiences that you think researchers or healthcare professionals should know?
2. Is there anything you think patients and their families should know?
3. Is there anything else you would like to share today?

**Thank you for your sharing your time and your experiences with me. We are now at the end of the discussion, and I will stop the recording.**
